# F_ST_-Based Marker Prioritization Within Quantitative Trait Loci Regions and Its Impact on Genomic Selection Accuracy: Insights from a Simulation Study with High-Density Marker Panels for Bovines

**DOI:** 10.3390/genes16050563

**Published:** 2025-05-10

**Authors:** Sajjad Toghiani, Samuel E. Aggrey, Romdhane Rekaya

**Affiliations:** 1 Animal Genomics and Improvement Laboratory, Agricultural Research Service, USDA, Beltsville, MD 20705, USA; 2 Institute of Bioinformatics, The University of Georgia, Athens, GA 30602, USA; saggrey@uga.edu (S.E.A.); rrekaya@uga.edu (R.R.); 3 Department of Poultry Science, The University of Georgia, Athens, GA 30602, USA; 4 Department of Animal and Dairy Science, The University of Georgia, Athens, GA 30602, USA

**Keywords:** genomic selection, F_ST_ prioritization, high-density marker panels

## Abstract

Background/Objectives: Genomic selection (GS) has improved accuracy compared to traditional methods. However, accuracy tends to plateau beyond a certain marker density. Prioritizing influential SNPs could further enhance the accuracy of GS. The fixation index (F_ST_) allows for the identification of SNPs under selection pressure. Although the F_ST_ method was shown to be able to prioritize SNPs across the whole genome and to increase accuracy, its performance could be further improved by focusing on the prioritization process within QTL regions. Methods: A trait with heritability of 0.1 and 0.4 was generated under different simulation scenarios (number of QTL, size of SNP windows around QTL, and number of selected SNPs within a QTL region). In total, six simulation scenarios were analyzed. Each scenario was replicated five times. The population comprised 30K animals from the last 2 generations (G9–G10) of a 10-generation (G1–G10) selection process. All animals in G9–10 were genotyped with a 600K SNP panel. F_ST_ scores were calculated for all 600K SNPs. Two prioritization scenarios were used: (1) selecting the top 1% SNPs with the highest F_ST_ scores, and (2) selecting a predetermined number of SNPs within each QTL window. GS accuracy was evaluated using the correlation between true and estimated breeding values for 5000 randomly selected animals from G10. Results: Prioritizing SNPs using F_ST_ scores within QTL window regions increased accuracy by 5 to 18%, with the 50-SNP windows showing the best performance. Conclusions: The increase in GS accuracy warrants the testing of the algorithm when the number and position of QTL are unknown.

## 1. Introduction

Genomic selection (GS) has become the standard tool for genetic evaluation due to its increased accuracy and substantial reduction in generation interval [1,2,3,4]. As a result, marker information is now systematically used to estimate genomically enhanced breeding values (GEBV) for livestock and poultry species. Recent developments in bovine genomics have led to the widespread use of both high-density (HD) and low- to moderate-density SNP panels for genomic selection. Commercially available panels, such as the Illumina BovineSNP50 BeadChip, with over 53,000 markers, and the BovineHD BeadChip, with up to 777,000 markers, provide genome-wide coverage for major dairy and beef breeds. Continuous improvement in high-throughput technologies and the dramatic decrease in genotyping and sequencing costs have led to a substantial increase in the density of the marker information and the number of genotyped animals [5].

This rapid decline in high-density SNP panels or whole-genome sequencing costs is poised to revolutionize genomic selection practices. As sequencing becomes increasingly affordable, the direct utilization of sequence-level data, capturing the full spectrum of genetic variation, is anticipated to supersede reliance on SNP panels. This shift promises to improve the resolution and accuracy of genomic predictions, accelerate the identification of candidate quantitative trait loci (QTL), and enable more precise selection decisions [6,7,8,9].

An immediate and significant challenge in the field of GS is the proliferation of genotyped variants, both rare and common, attributed to the notable advancement in next-generation sequencing. However, the gain in prediction accuracy seems to plateau after a certain marker density. This is partly because, once sufficient linkage disequilibrium (LD) between markers and QTL is captured, additional markers provide diminishing returns in terms of accuracy [10]. The optimality of HD SNP marker panels or even of widely used low–moderate commercial chips on GS accuracy have received little attention. Using all the SNPs in a marker panel is not necessarily the best option [4,11,12], and subsets of well-prioritized SNPs could lead to a significant increase in accuracy, as shown by [13,14,15]. Traditionally, variant prioritization relied on statistical criteria such as *p*-values for single marker analyses or on the quality of fit and model determination for Bayesian procedures like BayesB [16] and BayesR [17]. However, our research studies over the years have clearly indicated that exclusive reliance on statistical criteria may not be enough to prioritize influential variants and an alternative approach based on the fixation index was proposed [13,14,15,18,19]. Fixation index (F_ST_) scores, a measure of allele frequency differentiation among populations, can be used to pinpoint genome regions under selection pressure. These regions are likely to be areas harboring QTL-affecting traits under selection and their linked markers. In this study, a simulation was conducted to investigate the impact of different F_ST_ prioritization strategies on estimating the heritability and accuracy of GS under different heritability and complexity of the genetic model (number and size of QTL effects). These strategies were compared with the case when all HD SNP data were used.

## 2. Materials and Methods

### 2.1. Simulation: Population Structure

The QMSim software (version 2.0) [20] was used for data simulation. A quantitative trait with low (0.10) or moderate (0.40) heritability was simulated. All the genetic variance was assumed to be explained by the simulated QTL. The phenotypic variance was set to 1.00, and the residual variance was adjusted accordingly to get the desired heritability. A historical population of 5000 animals decreased to 400 after 1000 generations and gradually increased to 50,000 animals at generation 1300 to initialize LD and mutation–drift equilibrium. The founder population (G0) was generated by the random mating of non-selected 100 males to 15,000 females. Ten selection generations (G1–G10) of 15,000 animals each were simulated, and parent selection for each generation was based on estimated breeding values (EBVs) calculated using BLUP internally through the simulation. The replacement rate was 50% and 30% for males and females, respectively. The sex ratio in the progeny was maintained at 50% and one progeny per dam was assumed throughout. Only animals in the training (G9) and validation (G10) populations were genotyped. The true breeding value (TBV) of an individual was obtained as the sum of the QTL additive effects. Two fixed effects with 100 and 4 levels were simulated separately. The trait phenotypes were generated by adding the fixed effects, the TBV and random residual terms generated from a normal distribution with zero mean and variance equal to the residual variance. Table 1 provides a detailed description of the simulation process of the population structure. Parameter files for QMSim for each simulation scenario are provided in Supplementary Files S1–S6. The corresponding seed files for each simulation scenario are provided in Supplementary Files S7–S12. Supplementary Files S1–S4 are associated with simulations including 500 QTL, whereas Supplementary Files S5–S6 pertain to simulations with 2000 QTL. The seed files for these simulations are provided in Supplementary Files S7–S12, respectively.

### 2.2. Genome Structure

The simulated genome consisted of 29 autosomal chromosomes with a length of 2319 centimorgans (cM) to mimic a real bovine genome. Simulating the same number of autosomes with lengths identical to those of the real genome creates a more realistic scenario in terms of the number of physically unlinked markers and QTL loci. A total of 600K SNP markers with a minor allele frequency greater than 0.05 were randomly distributed throughout the genome. To test the impact of the genetic complexity of the trait, 500 and 2000 QTL were simulated with effects sampled from a normal distribution scaled appropriately to reflect the assumed genetic variance. A recurrent mutation rate of 2.5 × 10^−5^ was set for both markers and QTL to ensure mutation-drift equilibrium in historical generations and all subsequent selection generations. The same initial 0.5 allelic frequencies were assumed for both SNP markers and QTL in the historical population. All SNP markers and QTL were segregating in the last historical population and assumed to be bi-allelic in all simulated scenarios where no marker loci overlapped with the QTL. The simulation parameters used in generating the genome are presented in Table 1.

### 2.3. SNP Prioritization Method: F_ST_ Approach

Fixation indexes, particularly F_ST_, introduced by Wright [21], measure genetic differentiation through changes in allele frequencies among populations. The global F_ST_ estimator developed by Nei [22] was utilized in this study to prioritize SNPs, as proposed by Toghiani et al. [13] and Chang et al. [18]. Briefly, animals genotyped in generation 9 (G9; training population) were divided into two sub-populations based on the distribution of the trait phenotype (below the 5% quantile [SP1; bottom phenotype] and above the 95% quantile [SP2; top phenotype]). To calculate F_ST_ scores, the global F_ST_ estimator was utilized with subpopulations SP1 and SP2. The estimator is defined for a given locus (k) as follows:

 **(1)** $$F_{{ST}_{k}}=\frac{H_{T_{k}}-H_{{SW}_{k}}}{H_{T_{k}}}\;\mathrm{with}\;{H_{SW}}_{k}=\frac{{H_{S1}}_{k}*n_{s1}+{H_{S2}}_{k}*n_{s2}}{n_{s1}+n_{s2}},\;{H_{T}}_{k}=2*p_{k}*q_{k}\;\mathrm{and}\;{H_{Si}}_{k}=2*p_{{Si}_{k}}*q_{{Si}_{k}}$$

where $p_{{Si}_{k}}$ and $q_{{Si}_{k}}$ are the allele frequencies for locus k in subpopulation i of locus k, $n_{s1}$ and $n_{s2}$ are the number of individuals in the SP1 and SP2 subpopulations, ${H_{SW}}_{k}$ is the weighted mean heterozygosity across the SP1 and SP2 subpopulations, and ${H_{T}}_{k}$ is the heterozygosity of the pooled subpopulations for locus k. Theoretical methods are available for detecting loci under selection pressure using F_ST_ values, but their conservative approach may limit the predictive power of the selected list of SNPs. Toghiani et al. [13] heuristically used the top 97.5 or 99.5% quantiles of the F_ST_ distribution to prioritize SNPs in HD marker panels. Using these fixed thresholds yielded reasonable results in tracking genome regions under selection [13,18]. However, the F_ST_ method performance could be increased by (1) focusing the prioritization effort within areas of the genome with high probability of harboring QTL, and (2) reducing the number of SNP prioritized within a QTL window region. When using HD SNP panels, QTL, particularly those with moderate to high effects, induce a spike in the F_ST_ scores in proximal SNPs that frequently exhibit high LD both among themselves and with the casual QTL. Prioritizing only a small subset of these SNPs will be advantageous. To reach these goals, a new approach and algorithm to enhance the performance of the F_ST_ for SNP prioritization is proposed and consists of the following steps:

(1) Global F_ST_ scores are calculated for all 600K SNPs in the panel using Equation **(1)** and the 25% quantile of F_ST_ score distribution is determined as the global threshold point. To justify this specific threshold, a preliminary grid search across a range of FST quantile values was conducted. This analysis revealed that using the 25% quantile allowed for the identification of genomic windows encompassing approximately 99% of the simulated QTL, which collectively accounted for 98% and 93% of the total genetic variance in the simulation for the 500 and 2000 simulated QTL scenarios, respectively.
(2) The average F_ST_ scores for each window and QTL position is calculated (e.g., 50 SNP up and down stream of the QTL). This window-based averaging helped identify broader genomic regions under selection rather than relying on individual SNP scores.
(3) QTL regions with average window’s F_ST_ scores exceeding a defined threshold (e.g., average scores based on all 600K SNP markers) are qualified and retained. This step further refines the selection to focus on regions exhibiting strong signals of genomic differentiation.
(4) Within a window surrounding each retained QTL region, a small number of SNPs are randomly prioritized. The goal is to retain 1% (6000 SNPs) of the total SNPs in the panel for subsequent analyses.

To achieve such a target, 12 SNPs per window for the 500 QTL scenario and 3 SNPs per window for the 2000 QTL scenario were selected. This strategy aims to provide sufficient marker coverage within potentially important regions while adhering to our 1% prioritization goal. With 500 simulated QTL, selecting 12 SNPs per prioritized window contributed towards our 6000 SNP target. Similarly, with 2000 simulated QTL, selecting 3 SNPs per window allowed us to survey a broader range of potentially relevant regions while staying within our 1% prioritization constraint. To assess the impact of the proposed algorithm on the estimation of genetic parameters and the accuracy of genomic selection, several simulation scenarios were implemented. The simulation parameters considered in this study were heritability (0.1 and 0.4), number of QTL (**Q1** = 500 and **Q2** = 2000), windows size as indicated by the number of SNP markers up and downstream of the QTL (**W1** = 50, **W2** = 100, **W3** = 200, and **W4** = 400 markers), and number of SNP markers selected in each QTL window (**P1** = 3 SNPs and **P2** = 12 SNPs). For each heritability value, the following analysis scenarios were implemented: **FULL600K** (using all 600K SNP markers), **Top1%F_ST_** (top 1% of SNPs with the highest F_ST_ scores), **W1Q1P2** (Window 1 with 500 QTL and 12 selected SNPs within QTL window), **W2Q1P2** (Window 2 with 500 QTL and 12 selected SNPs within QTL window), **W3Q1P2** (Window 3 with 500 QTL and 12 selected SNPs within QTL window), **W4Q1P2** (Window 4 with 500 QTL and 12 selected SNPs within QTL window), **W1Q2P1** (Window 1 with 2000 QTL and 3 selected SNPs within QTL window), **W2Q2P1** (Window 2 with 2000 QTL and 3 selected SNPs within QTL window), **W3Q2P1** (Window 3 with 2000 QTL and 3 selected SNPs within QTL window), **and W4Q2P1** (Window 4 with 2000 QTL and 3 selected SNPs within QTL window)**.**

### 2.4. Statistical Model and Data Analysis

In all simulated data scenarios, 15,000 genotyped animals from G9 were used as the training population, while 5000 genotyped animals from G10 were randomly selected and used as the validation set. To compare the different F_ST_ prioritization scenarios described above, the following mixed model was used

 **(2)** y=Xb+Zu+e

where y is the vector of phenotypes, b is the vector of fixed effects, u is the vector of genomic breeding values, and e is the vector of random residuals. X and Z are known incidence matrices with the appropriate dimensions. Furthermore, it was assumed that $u\sim N(0,G\sigma_{u}^{2})$, where G is the genomic relationship matrix and $\sigma_{u}^{2}$ is the genetic variance. AIREMLF90 (version 1.148) and BLUPF90 (version 1.71) programs [23] were used for the implementation of the model in Equation **(2)**. The accuracy of genomic evaluation was assessed by calculating the correlation between the true and estimated genomic breeding values in the validation population. Each simulation scenario was replicated 5 times.

## 3. Results

### 3.1. Detected QTL and Their Contribution to the Total Genetic Variance

Table 2 presents the means and standard deviations of allelic substitution effects and the percentage of genetic variance contribution across distinct QTL categories for a trait with heritability of 0.4. Under the 500 QTL scenario, the upper 5% quantile of QTL accounted for approximately 26.6% of the total genetic variance (GV). The QTL, within the 25 and 75% interquartile range, contributed a comparable proportion (25.8%) to GV. In contrast, QTL in the lower 5% quantile exhibited minimal influence, accounting for less than 0.1% of the total GV. The 6253-fold difference in contribution to the GV observed between the top and bottom 5% QTL groups (1.063% vs. 0.00017%) highlights the substantial heterogeneity of the trait genetic architecture. The allelic substitution effects showed a consistent and expected scaling pattern, being around 50% smaller when the number of QTL quadrupled from 500 to 2000 (from 0.1056 to 0.0515 for the top 5% QTL). Furthermore, the standard deviations for both allelic substitution effects and variance explained decreased progressively from the top to bottom QTL categories, suggesting greater homogeneity among smaller-effect QTL. The observed patterns of contribution to the genetic variance were maintained in the 2000 QTL scenario, although the per-QTL contribution was proportionally reduced across all categories. This consistent dilution of QTL effects suggests that the architecture of the trait has become more diffuse as the trait becomes more polygenic. Analogous results for when the heritability was equal to 0.1 are presented in Supplementary Table S1.

### 3.2. Exploring F_ST_ Score Patterns Across QTL Effect Classes

A summary description of the distribution of F_ST_ scores for SNPs surrounding QTL with different contributions to the total genetic variance across different simulation scenarios when the heritability was equal to 0.4 is presented in Table 3. Corresponding results for when heritability was equal to 0.1 are provided in Supplementary Table S2. Across the different SNP windows (**W1** to **W4**) and number of QTL (**Q1** = 500 or **Q2** = 2000) scenarios, the average F_ST_ scores decayed, as expected, with the decrease in the QTL effects. In fact, the average F_ST_ scores for large-effect QTL (95% quantile) were consistently higher than those for medium and small-effect QTL across all scenarios. This pattern was most pronounced in narrower SNP windows (**W1**), where the largest-effect QTL showed approximately 1.5 times higher F_ST_ scores compared to small-effect QTL. As window size increased from **W1** to **W4**, this differentiation became less pronounced. Additionally, scenarios with fewer QTL (**Q1** = 500) exhibited slightly stronger differentiation between large and small effect sizes compared to scenarios with more QTL (**Q2** = 2000).

**Figure 1:**
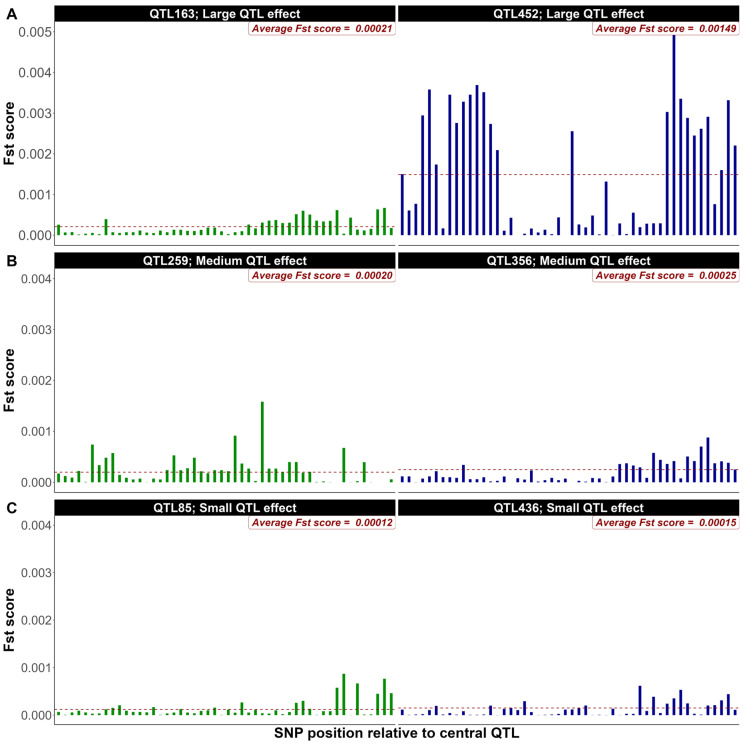
F_ST_ score distribution for 50-SNP windows surrounding large (**A**), medium (**B**), and small (**C**) QTL selected based on their allelic substitution effects (shown as an example from the first replicate of simulated data with 500 QTL and heritability of 0.4). The green and blue bars represent the F_ST_ score for each SNP within the 50-SNP windows surrounding two QTL selected from each group (large, medium and small). The red horizontal dashed lines indicate the average F_ST_ scores within the 50-SNP windows.

**Figure 2:**
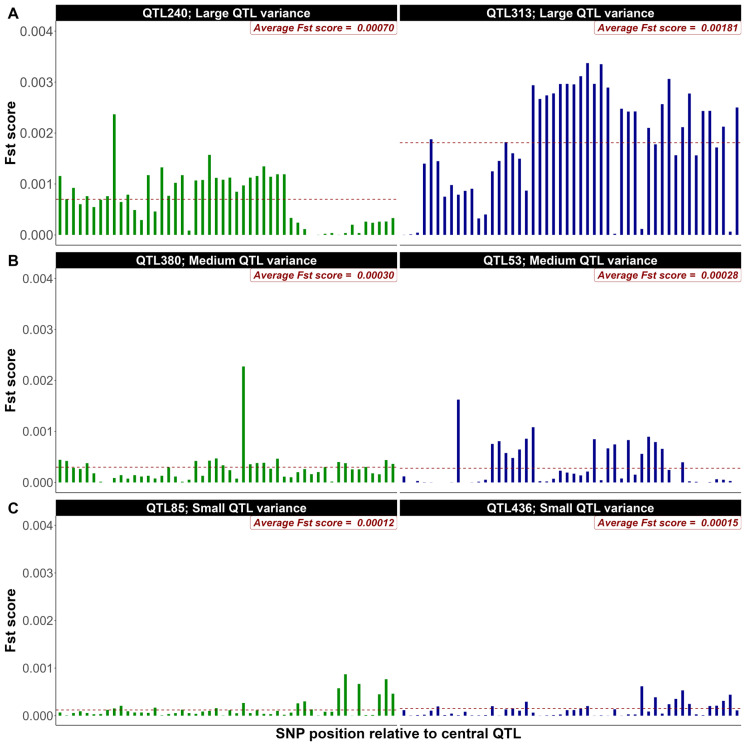
F_ST_ score distribution for 50-SNP windows surrounding large (**A**), medium (**B**), and small (**C**) QTL selected based on their contribution to the total genetic variance (Shown as an example from the first replicate of simulated data with 500 QTL and heritability of 0.4). The green and blue bars represent the F_ST_ score for each SNP within the 50-SNP windows surrounding two QTL selected from each group (large, medium and small). The red horizontal dashed lines indicate the average F_ST_ scores within the 50-SNP windows.

As illustrative examples from the first replicate of simulated data (500 QTL, heritability 0.4), Figure 1 and Figure 2 present the distribution of F_ST_ scores for a 50-SNP window equally distributed up and downstream of the QTL (**W1** = 50 SNPs) for two large, medium, and small ranked QTL based on their allelic effects (Figure 1) and percentage of genetic variance explained (Figure 2), respectively. As expected, the distribution of F_ST_ scores showcases higher average scores for large-effect QTL compared to their medium and small-effect counterparts. F_ST_ scores seem to be higher when QTL effects were assessed based on their contribution to the genetic variance (Figure 2). These figures highlight the role of QTL effects in shaping genetic variation within a population and clearly illustrate the variation in F_ST_ scores within and across QTL effect classes.

### 3.3. Genomic Predictions Across the Different Simulation Scenarios

Table 4, Table 5, Table 6 and Table 7 present the average number of SNPs, variance component estimates, heritability, and genomic prediction accuracy across different simulation parameters based on five replicates. Variance component and heritability estimates were almost identical to the true values when heritability was set to 0.1 (Table 5 and Table 7) and slightly lower when the true heritability was 0.4 (Table 4 and Table 6). For both heritability scenarios, a substantial underestimation of the genetic variance and heritability, coupled with an overestimation of the residual variance, was observed, particularly in the **Top1%F_ST_** scenario, when only 1% of the SNPs were prioritized based on their individual F_ST_ scores. Except for the **Top1%F_ST_** scenario, all other SNP prioritization scenarios considered in this study increased genomic prediction accuracy (Table 4, Table 5, Table 6 and Table 7).

Furthermore, across all scenarios excluding **Top1%F_ST_**, most of the QTL regions were successfully identified, as judged by average F_ST_ scores for SNPs in the window surrounding the QTL exceeding the first quartile of the genome-wide F_ST_ score distribution. Consequently, nearly all the genetic variance was explained by the selected QTL regions. In fact, the number of detected QTL ranged between 493 and 500 and between 1975 and 2000 for the 500 QTL and 2000 QTL simulation scenarios, respectively. The percentage of genetic variance explained by the identified QTL ranged between 94.12 and 96.87% across the different simulation scenarios.

## 4. Discussion

### 4.1. Detected QTL and Their Contribution to the Total Genetic Variance

When simulated QTL were clustered into groups based on the distribution of their effects for a trait with heritability of 0.4 (Table 2), the percentage variance explained approach was more efficient compared to the allele substitution effect in separating QTL groups. This aligns with classical quantitative genetics, where the variance explained by a QTL depends on both its effect size and allele frequency, and is a more direct measure of its contribution to trait variation [24]. In fact, the average variance explained by a Top 5% QTL (1.06%) under the 500 QTL simulation scenario is more than 10-fold greater compared to the average of a medium size QTL (0.103). Using the allele substitution effects, the same comparison resulted in only a 3-fold change advantage for the Top 5% group (Table 2). Across the different simulation scenarios, the bottom 5% QTL had practically no contribution to the total genetic variance, consistent with theoretical expectations that small-effect QTL contribute little to overall trait variance [24]. Trying to track these and other small effect QTL will have a negative effect on accuracy due to decreased QTL similarity between animals. In simulated as well as with real data, the number and distribution of QTL effects (indicator of the genetic complexity of a trait) play a major role in the efficiency of a marker prioritization approach and its impact on the accuracy of genomic predictions. Thus, the potential benefits of a prioritization approach are largely trait specific. The decay of average F_ST_ scores with decreasing QTL effects, was as expected with larger-effect QTL exerting a stronger selective pressure and could lead to greater divergence between subpopulations (Table 3). The more pronounced differentiation between large- and small-effect QTL in narrower SNP windows (**W1**) suggests that the strongest selection signatures are concentrated near the causal variant (Table 3). The dilution of the QTL effect, seen as the window size increased from **W1** to **W4,** indicates that including more distant SNPs weakens the signal, likely due to reduced linkage disequilibrium (LD). The slightly stronger differentiation observed in scenarios with fewer QTL (**Q1** = 500) compared to those with more QTL (**Q2** = 2000) suggests that the underlying genetic architecture influences the detectability of selection signatures.

### 4.2. Exploring FST Score Patterns Across QTL Effect Classes

When QTL were ranked based on their allelic substitution effects for a heritability of 0.4 and 500 QTL in Figure 1, the average F_ST_ scores of the 50 SNPs surrounding two large QTL (Figure 1A) was 0.57 (QTL 163) to 4 (QTL 452) folds greater that the genome wide average F_ST_ scores (0.00037). Similarly, the average F_ST_ scores of the 50 surrounding SNPs were 0.54 to 0.67 and 0.32 to 0.40 folds greater than the genome-wide average for medium (Figure 1B) and small (Figure 1C) QTL effects, respectively. In fact, there were marked differences in the average and distribution of the F_ST_ scores even for QTL within each of the three effect classes. Furthermore, some SNPs surrounding medium or even small effect QTL had higher individual F_ST_ scores than some of those surrounding large effect QTL (Figure 1). This is due, as expected, to the variation in minor allele frequencies between these SNPs. More problematic is the similarity in the mean of F_ST_ scores between SNPs surrounding QTL with different effect sizes (QTL 163, Figure 1A(left); QTL 259, Figure 1B(left)). Upon ranking QTL based on their relative contribution to the genetic variation of the trait in Figure 2, more pronounced differences in the distribution and average F_ST_ scores were observed across various QTL effect classes compared to rankings based on allelic substitution effects. Additionally, there was no overlap observed between QTL of different size classes. In both scenarios, F_ST_ score averages and distributions induced by small QTL were not significantly different from the background genome. Similar trends were identified when the heritability or the number of QTL was set to 0.1 and 2000, respectively. Collectively, these results seem to indicate that SNPs are more efficiently prioritized based on their association with the genetic variance rather than their allelic substitution effect explained by the QTL. Using *p*-values or estimates of SNP effects from single or joint marker analyses will suffer from high false positives, multiple testing problems, high LD [25,26]. In contrast, the F_ST_ approach intrinsically prioritizes SNPs linked to QTL with significant contribution to the genetic variance through allele frequency divergence. This could explain in part the superiority of the F_ST_ approach compared to other methods [13,14,18]. QTL with small effects are unlikely to be tracked with a group of surrounding SNPs due to their very insignificant impact on the spike of F_ST_ scores (Figure 1C and Figure 2C). On the other hand, the joint contribution of these small QTL to the genetic variance is often small. In fact, the bottom 25% of QTL simulated in our study explained only 0.89% of the total genetic variance. Similar results were observed for the other simulation scenarios.

### 4.3. Genomic Predictions Across the Different Simulation Scenarios

The genomic prediction accuracy depends on several factors including the size and structure of the training population, the trait heritability, the density of the SNP markers, the quality of the dependent variable and genomic information, the genetic relatedness between training and validation sets, the LD between marker and QTL, and the effective population size [27,28,29]. Using all SNPs in the panel (**FULL600K**) when the simulated trait was controlled by 500 QTL (Table 4 and Table 5), almost 97% of the genetic variance was captured regardless of the heritability level. The remaining 3% of the genetic variance was not captured due to the fixation of some QTL during the simulation process. As expected, the highest percentage of GV explained was achieved when the genomic relationship matrix (**G**) used in the GBLUP analysis was calculated based on **FULL600K** scenario. Using the prioritized 6000 SNPs based on the highest F_ST_ scores across the genome to compute **G** resulted in an underestimation and an overestimation of the genetic and residual variances, consequently leading to a substantial underestimation of the heritability. In fact, the estimated heritability values are notably lower, showing a reduction of 30 and 40% compared to the estimates derived from **FULL600K** scenario when the true heritability was set at 0.4 and 0.1, respectively (Table 4 and Table 5). Similarly, there was a 22 and 27% decrease in the accuracy of the predicted genomic breeding values. The decrease in heritability estimates and prediction accuracy under the **Top1%F_ST_** scenario can be attributed largely to the limited ability of the small number of prioritized SNPs to track most of the QTL. When 12 SNPs (**P2**) within a window of 50 (**W1**) to 400 (**W4**) markers surrounding a QTL and 500 simulated QTL (**Q1** = 500) were randomly selected (e.g., **W1Q1P2**)**,** 5906 to 5964 and 5887 to 5957 markers were prioritized for heritability equal to 0.4 and 0.1, respectively, across different QTL regions. Across various windows (**W1**–**W4**) and heritabilities (0.4 and 0.1), over 95% of the GV was captured, with the maximum difference being less than 1.5% compared to using all 600 markers (refer to Table 4 and Table 5). Using these prioritized SNPs to compute **G** resulted only in slightly lower estimates of GV and heritability compared to when all 600K SNPs were used. These findings indicate that prioritizing around 1% of SNPs within QTL regions, rather than across the entire genome as in the **Top1%F_ST_** scenario, is sufficient to yield similar genetic parameter estimates to the **FULL600K** scenario. Furthermore, F_ST_-based SNP prioritization within QTL regions resulted in increased accuracy ranging between 3% to 18% and 1% to 14% for heritabilities of 0.1 and 0.4, respectively. The findings, although slightly different in magnitude, are in line with those reported by Chang et al. [15]. This is likely due to the increased relative weight of SNPs associated with large and moderate QTL in the calculation of **G** [15]. For both heritabilities, the maximum accuracy was achieved when the QTL window consisted of 50 SNPs and decreased with the increase in the window size (Table 4 and Table 5). Furthermore, there is no need to prioritize every SNP within a QTL region.

**Figure 3:**
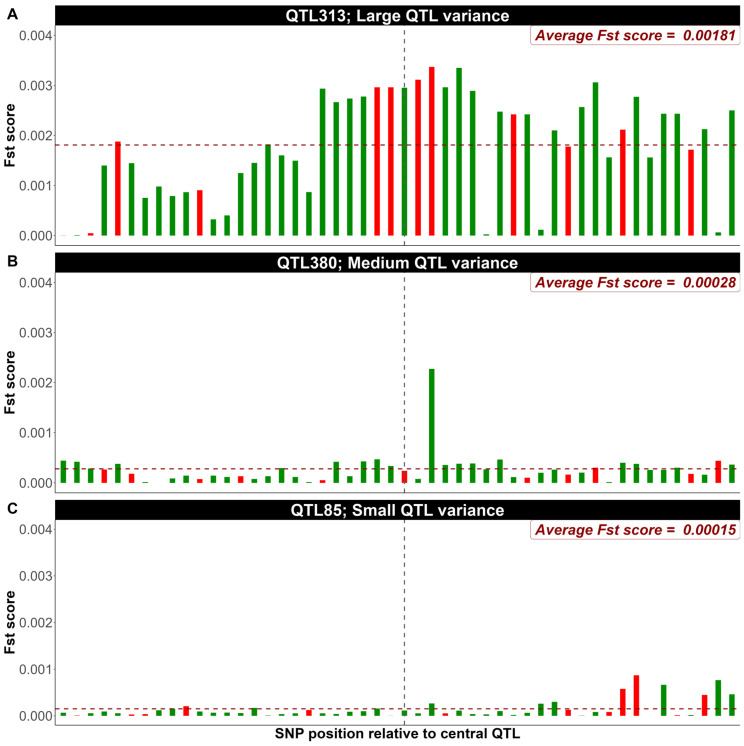
Distribution of F_ST_ scores within a 50-SNP window surrounding individual QTL with large (**A**), medium (**B**), and small (**C**) contributions to the genetic variance. Green bars represent the F_ST_ score for each SNP within the 50-SNP window, and red bars represent the position and F_ST_ scores of the randomly selected 12 SNPs. The vertical and horizontal dashed lines indicate the QTL position and the average F_ST_ scores within the window, respectively (shown as an example from the first replicate of simulated data with 500 QTL and heritability of 0.4).

**Figure 4:**
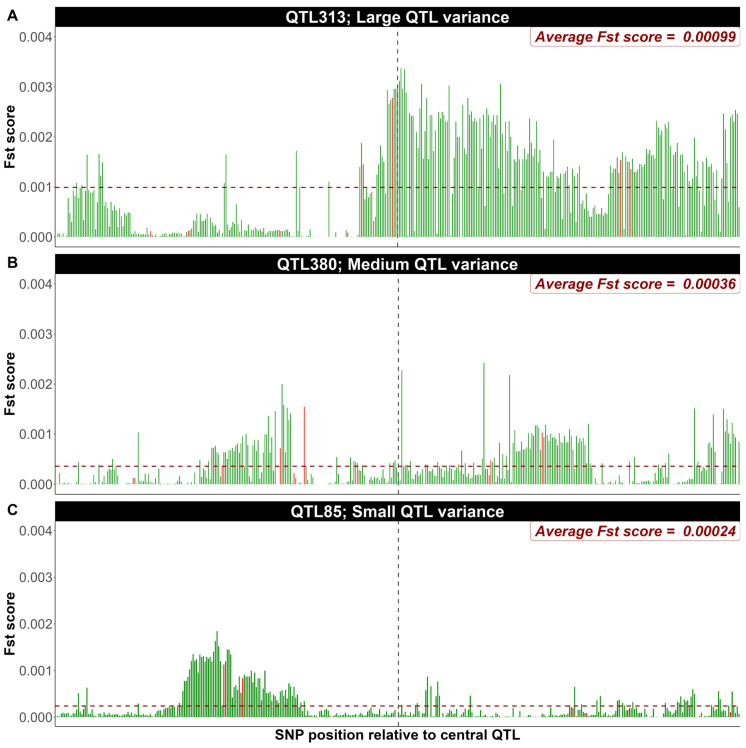
Distribution of F_ST_ scores within a 400-SNP window surrounding individual QTL with large (**A**), medium (**B**), and small (**C**) contributions to the genetic variance. Green bars represent the F_ST_ score for each SNP within the 400-SNP window, and red bars represent the position and F_ST_ scores of the randomly selected 12 SNPs. The vertical and horizontal dashed lines indicate the QTL position and the average F_ST_ scores within the window, respectively (shown as an example from the first replicate of simulated data with 500 QTL and heritability of 0.4).

In fact, a randomly selected small number of SNPs within a QTL region (e.g., 12 markers) is sufficient to track the QTL effect. This is the case due to the high LD among SNPs within a QTL window. As an example from the first replicate of simulated data (500 QTL, heritability 0.4), a closer inspection of F_ST_ score distributions within 50-SNP (Figure 3) and 400-SNP windows (Figure 4) surrounding individual QTL with large, medium and small genetic variance contribution reveals, as expected, that the spikes in F_ST_ scores decay monotonously as the distance from the QTL increases. Randomly selecting 12 SNPs within each window surrounding QTL region, expanding the window increases the probability of incorporating SNPs with lower LD with the QTL as shown in Figure 4. Based on the SNP marker density used in this study, randomly prioritizing around 25% (12 SNPs) of markers within a 50-SNP window appears to provide the optimal configuration for capturing stronger F_ST_ signals in proximity to causal variants.

When the trait was controlled by 2000 QTL, only 3 SNPs were randomly prioritized within each window, maintaining an equivalent number of selected markers (6000 SNPs) in comparison to the scenarios with 500 QTL. Overall, the trends and magnitudes of the results closely mirrored those observed in the 500 QTL scenarios as indicated in Table 6 and Table 7. In fact, when the heritability was equal to 0.10, 93 to 94% of the GV was tracked. As with the 500 QTL scenarios, the maximum accuracy was achieved when the window size consisted of 50 SNPs surrounding the QTL. The genomic prediction accuracy was 0.78 and 0.64 using all SNPs (**FULL600K**) for heritability of 0.4 and 0.1, respectively, and it increased by 2–6% (Table 6) and 1–5% (Table 7) when using prioritized SNPs within QTL regions. This increase in accuracy seems to stem from achieving a balance between the percentage of genetic variance explained by the selected SNPs and the resulting genetic similarity among individuals based on those markers, as shown by Toghiani et al. [13]. Opting for the smallest SNP window surrounding QTL increases the likelihood of selecting SNPs in high LD with the QTL based on their F_ST_ scores, which results, ultimately, in enhanced genomic accuracy.

This study primarily focused on the potential for increased prediction accuracy through targeted SNP prioritization using the F_ST_ approach. However, the importance of quantifying computational resources is acknowledged, as computational efficiency is a key advantage of reducing marker dimensionality. In this initial simulation study, memory usage and CPU time were not explicitly measured and compared. Nonetheless, the rationale for emphasizing lower computational cost is directly related to the substantial reduction in the number of SNPs used in the genomic prediction models. The prioritization strategy, retaining only approximately 1% of the original 600K markers, is expected to significantly decrease the computational demands of downstream analyses, including model training and prediction, due to the reduced data dimensionality and fewer parameters to estimate.

## 5. Conclusions

The results of this study clearly highlight the possibility of further increasing the accuracy of genomic selection through the prioritization of a small subset of relevant SNPs to calculate **G**. The F_ST_-based SNP prioritization approach was efficient in tracking the most influential QTL. Focusing on SNPs within 50-SNP windows surrounding QTL seems to be the optimum setup. Based on our simulation parameters, a 50-SNP window spans an average of 186.7 kbp. For different genomes and marker panel density, these parameters can be used to approximate the window size. Our findings highlight the importance of focusing on genome regions under selection pressures, leading to more accurate genetic evaluations and improved GS accuracy. The demonstrated efficacy of the F_ST_-based approach to prioritize SNPs within QTL regions supports its potential to significantly enhance the efficiency of breeding programs. The proposed approach contributes to the enhancement and fine-tuning of genomic selection tools and techniques, emphasizing the need for future research to explore its wide-ranging application in different livestock species and complex traits. Investigating the intricate dynamics between F_ST_ score distribution, QTL density, trait complexity, and distribution of marker effects will be crucial in optimizing SNP prioritization strategies. Such efforts will help develop more precise, efficient, and cost-effective genomic selection methods for the livestock industry.

## Figures and Tables

**Table 1:** Simulation parameters used for population and genomic structures.

Population Structure	
**Step 1: Historical generations (HG)**	
Size of HG [number of generations]	5000[0] 400[1000] 50,000[1300]
**Step 2: Recent generations**	
Founder male selected from HG	100
Founder female selected from HG	15,000
Number of offspring per dam	1
Mating design	random
Selection design	EBV
EBV estimation method	BLUP animal model
Sex ratio	0.50
Sire replacement rate	0.50
Dam replacement rate	0.30
Number of generations	10
Genotyped generations	9, 10
Heritability of trait	0.40, 0.10
Phenotypic variance	1
**Genomic structure**	
Number of Chromosomes	29
Total Chromosome length	2319 cM
Number of SNP markers	600K SNP
Marker distribution	Evenly spaced
Number of QTL	500, 2000
QTL distribution	Random
MAF threshold for markers and QTL	0.05
QTL allele effects	Normal distribution
Marker and QTL recurrent mutation	2.5 × 10^−5^

**Table 2:** Summary statistics of allelic substitution effects and genetic variance contribution for selected QTL within different QTL groups (heritability = 0.4).

QTL Group ^1^	# Simulated QTL	# Selected QTL	Allele Substitution		Variance Explained (%)	
			Mean	SD	Mean	SD
**Top 5%**	500	25	0.1056	0.0163	1.063	0.338
	2000	100	0.0515	0.0076	0.283	0.116
**Q25_Q75**	500	250	0.0315	0.0107	0.103	0.065
	2000	1000	0.0156	0.0052	0.024	0.016
**Bottom 5%**	500	25	0.0013	0.0007	0.00017	0.00015
	2000	100	0.0008	0.0005	0.00006	0.00006

^1^ **Top 5%** = the 5% QTL with the largest effects; **Q25_Q75** = all QTL with effect between the 25 and 75% quantiles of the effect distribution; and **Bottom 5%** = the 5% QTL with the smallest effects.

**Table 3:** Mean and standard deviation of FST scores for SNP windows surrounding QTL categorized by genetic variance contribution across different simulation scenarios (heritability = 0.40; first replicate).

Scenarios ^1^	Large QTL		Medium QTL		Small QTL	
	95% Quantile ^2^		25–75% Quantiles		5% Quantile	
	Mean	SD	Mean	SD	Mean	SD
**W1Q1**	0.00056	0.00043	0.00037	0.00031	0.00038	0.00034
**W2Q1**	0.00052	0.00048	0.00037	0.00036	0.00041	0.00037
**W3Q1**	0.00045	0.00046	0.00037	0.00041	0.00040	0.00043
**W4Q1**	0.00038	0.00044	0.00037	0.00045	0.00038	0.00046
**W1Q2**	0.00050	0.00040	0.00042	0.00035	0.00041	0.00037
**W2Q2**	0.00049	0.00046	0.00041	0.00041	0.00040	0.00039
**W3Q2**	0.00045	0.00049	0.00041	0.00046	0.00041	0.00045
**W4Q2**	0.00045	0.00053	0.00042	0.00051	0.00042	0.00049

^1^ **Window size: W1** = 50 SNPs, **W2** = 100 SNPs, **W3** = 200 SNPs, and **W4** = 400 SNPs; total number of QTL: **Q1** = 500 QTL, and **Q2** = 2000 QTL; ^2^ QTL effects were categorized based on their contribution to the genetic variance.

**Table 4:** Number of selected QTL and SNPs, percentage of genetic variance explained (%GV), variance component and heritability estimates, and accuracy of genomic prediction under different simulation scenarios with 500 QTL and heritability of 0.4.

Scenarios ^1^	#QTL	# SNPs	%GV	VG	VE	h^2^	Accuracy
**FULL600K**	500	600K	96.86	0.35	0.61	0.37 (0.005)	0.77 (0.006)
**Top1%F_ST_**	-	6000	-	0.27	0.76	0.26 (0.014)	0.60 (0.020)
**W1Q1P2**	496	5906	95.91	0.32	0.62	0.34 (0.004)	0.88 (0.004)
**W2Q1P2**	498	5949	96.48	0.32	0.62	0.34 (0.005)	0.85 (0.002)
**W3Q1P2**	499	5964	96.45	0.32	0.63	0.34 (0.005)	0.82 (0.004)
**W4Q1P2**	498	5950	96.36	0.31	0.65	0.32 (0.002)	0.78 (0.006)

^1^ **Full600K:** All 600K SNPs; **Top1%F_ST_**: Top 1% SNPs with the highest F_ST_ scores; **W*i*Q1P2**: 12 randomly prioritizing SNPs within a window *i* (**W1** = 50 SNPs, **W2** = 100 SNPs, **W3** = 200 SNPs, and **W4** = 400 SNPs) and **Q1** = 500 QTL. Standard errors are listed between parentheses. Results are based on the average of 5 replicates.

**Table 5:** Number of selected QTL and SNPs, percentage of genetic variance explained (%GV), variance component and heritability estimates, and accuracy of genomic prediction under different simulation scenarios with 500 QTL and heritability of 0.1.

Scenarios ^1^	#QTL	# SNPs	%GV	VG	VE	h^2^	Accuracy
**FULL600K**	500	600K	96.87	0.10	0.90	0.10 (0.004)	0.66 (0.007)
**Top1%F_ST_**	-	6000	-	0.06	0.94	0.06 (0.002)	0.48 (0.013)
**W1Q1P2**	493	5887	95.47	0.09	0.90	0.09 (0.002)	0.78 (0.007)
**W2Q1P2**	498	5944	96.47	0.09	0.90	0.09 (0.002)	0.75 (0.007)
**W3Q1P2**	499	5957	96.65	0.09	0.91	0.09 (0.002)	0.71 (0.007)
**W4Q1P2**	498	5950	96.63	0.09	0.91	0.09 (0.002)	0.68 (0.009)

^1^ **Full600K:** All 600K SNPs; **Top1%F_ST_**: Top 1% SNPs with the highest F_ST_ scores; **W*i*Q1P2**: 12 randomly prioritizing SNPs within a window *i* (**W1** = 50 SNPs, **W2** = 100 SNPs, **W3** = 200 SNPs, and **W4** = 400 SNPs) and **Q1** = 500 QTL. Standard errors are listed between parentheses. Results are based on the average of 5 replicates.

**Table 6:** Number of selected QTL and SNPs, percentage of genetic variance explained (%GV), variance component and heritability estimates, and accuracy of genomic prediction under different simulation scenarios with 2000 QTL and heritability of 0.4.

Scenarios ^1^	#QTL	# SNPs	%GV	VG	VE	h^2^	Accuracy
**FULL600K**	2000	600K	96.69	0.36	0.60	0.37 (0.01)	0.78 (0.01)
**Top1%F_ST_**	-	6000	-	0.25	0.76	0.25 (0.007)	0.56 (0.02)
**W1Q2P1**	1976	5901	95.25	0.32	0.63	0.34 (0.004)	0.83 (0.01)
**W2Q2P1**	1995	5954	96.22	0.31	0.65	0.32 (0.004)	0.80 (0.01)
**W3Q2P1**	1997	5948	96.41	0.31	0.65	0.32 (0.003)	0.76 (0.01)
**W4Q2P1**	1991	5929	96.17	0.29	0.66	0.30 (0.003)	0.75 (0.01)

^1^ **Full600K:** All 600K SNPs; **Top1%F_ST_**: Top 1% SNPs with the highest F_ST_ scores; **W*i*Q1P1**: 3 randomly prioritizing SNPs within a window *i* (**W1** = 50 SNPs, **W2** = 100 SNPs, **W3** = 200 SNPs, and **W4** = 400 SNPs) and **Q1** = 2000 QTL. Standard errors are listed between parentheses. Results are based on the average of 5 replicates.

**Table 7:** Number of selected QTL and SNPs, percentage of genetic variance explained (%GV), variance component and heritability estimates, and accuracy of genomic prediction under different simulation scenarios with 2000 QTL and heritability of 0.1.

Scenarios ^1^	#QTL	# SNPs	%GV	VG	VE	h^2^	Accuracy
**FULL600K**	2000	600K	94.42	0.09	0.90	0.09 (0.004)	0.64 (0.004)
**Top1%F_ST_**	-	6000	-	0.05	0.95	0.05 (0.004)	0.43 (0.014)
**W1Q2P1**	1975	5888	93.17	0.08	0.91	0.08 (0.004)	0.67 (0.004)
**W2Q2P1**	1995	5955	94.12	0.08	0.91	0.08 (0.003)	0.65 (0.005)
**W3Q2P1**	1996	5957	94.28	0.08	0.91	0.08 (0.003)	0.63 (0.005)
**W4Q2P1**	1992	5943	94.07	0.08	0.92	0.08 (0.004)	0.62 (0.008)

^1^ **Full600K:** All 600K SNPs; **Top1%F_ST_**: Top 1% SNPs with the highest F_ST_ scores; **W*i*Q1P1**: 3 randomly prioritizing SNPs within a window *i* (**W1** = 50 SNPs, **W2** = 100 SNPs, **W3** = 200 SNPs, and **W4** = 400 SNPs) and **Q1** = 2000 QTL. Standard errors are listed between parentheses. Results are based on the average of 5 replicates.

## Data Availability

Our workflow and pipelines, along with supporting data and scripts to validate the results detailed in this article, are available in GitHub at https://github.com/stghn/Prioritized-Fst-HDsim-QTLregion (accessed on 22 March 2025). The scripts include a mixture of R, Fortran, and bash scripts essential for generating simulated data, data processing, and implementation of genomic predictions, leading to the creation of figures and tables presented in this manuscript. Furthermore, Supplementary Files containing simulation parameters and seeds are available for download from our GitHub repository.

## Supplementary Materials

The following supporting information can be downloaded at: https://www.mdpi.com/article/10.3390/genes16050563/s1, File S1. QMSim parameter file for first replicate of the simulated data for a trait with 500 QTL distributed across 29 autosomal chromosomes and a heritability of 0.10; File S2. QMSim parameter file for replicates 2-5 of the simulated data for a trait with 500 QTL distributed across 29 autosomal chromosomes and a heritability of 0.10; File S3. QMSim parameter file for first replicate of the simulated data for a trait with 500 QTL distributed across 29 autosomal chromosomes and a heritability of 0.40; File S4. QMSim parameter file for replicates 2-5 of the simulated data for a trait with 500 QTL distributed across 29 autosomal chromosomes and a heritability of 0.40; File S5. QMSim parameter file for all 5 replicates of the simulated data for a trait with 2000 QTL distributed across 29 autosomal chromosomes and a heritability of 0.10; File S6. QMSim parameter file for all 5 replicates of the simulated data for a trait with 2000 QTL distributed across 29 autosomal chromosomes and a heritability of 0.40; File S7. QMSim seed file for first replicate of the simulated data for a trait with 500 QTL distributed across 29 autosomal chromosomes and a heritability of 0.10; File S8. QMSim seed file for replicates 2-5 of the simulated data for a trait with 500 QTL distributed across 29 autosomal chromosomes and a heritability of 0.10; File S9. QMSim seed file for first replicate of the simulated data for a trait with 500 QTL distributed across 29 autosomal chromosomes and a heritability of 0.40; File S10. QMSim seed file for replicates 2-5 of the simulated data for a trait with 500 QTL distributed across 29 autosomal chromosomes and a heritability of 0.40; File S11. QMSim seed file for all 5 replicates of the simulated data for a trait with 2000 QTL distributed across 29 autosomal chromosomes and a heritability of 0.10; File S12. QMSim seed file for all 5 replicates of the simulated data for a trait with 2000 QTL distributed across 29 autosomal chromosomes and a heritability of 0.40; Table S1. Summary statistics of allelic substitution effects and genetic variance contribution for selected QTL within different QTL groups (heritability=0.1); Table S2. Mean and standard deviation of F_ST_ scores for SNP windows surrounding QTL categorized by genetic variance contribution across different simulation scenarios (heritability= 0.10)
