# Supplementary material for: FST-Based Marker Prioritization Within Quantitative Trait Loci Regions and Its Impact on Genomic Selection Accuracy: Insights from a Simulation Study with High-Density Marker Panels for Bovines
Source: Genes (Basel). 2025 May 10;16(5):563. doi: 10.3390/genes16050563 (PMC12111557; doi:10.3390/genes16050563)
Supplement: Supplementary file 1 [file genes-16-00563-s001.zip › Supplemental_Material_Legends_G3.pdf]

Supplemental Material Legends

File S1: QMSim parameter file for first replicate of the simulated data for a trait with 500 QTL distributed across 29 autosomal chromosomes and a heritability of 0.10

File S2: QMSim parameter file for replicates 2-5 of the simulated data for a trait with 500 QTL distributed across 29 autosomal chromosomes and a heritability of 0.10

File S3: QMSim parameter file for first replicate of the simulated data for a trait with 500 QTL distributed across 29 autosomal chromosomes and a heritability of 0.40

File S4: QMSim parameter file for replicates 2-5 of the simulated data for a trait with 500 QTL distributed across 29 autosomal chromosomes and a heritability of 0.40

File S5: QMSim parameter file for all 5 replicates of the simulated data for a trait with 2,000 QTL distributed across 29 autosomal chromosomes and a heritability of 0.10

File S6: QMSim parameter file for all 5 replicates of the simulated data for a trait with 2,000 QTL distributed across 29 autosomal chromosomes and a heritability of 0.40

File S7: QMSim seed file for first replicate of the simulated data for a trait with 500 QTL distributed across 29 autosomal chromosomes and a heritability of 0.10

File S8: QMSim seed file for replicates 2-5 of the simulated data for a trait with 500 QTL distributed across 29 autosomal chromosomes and a heritability of 0.10

File S9: QMSim seed file for first replicate of the simulated data for a trait with 500 QTL distributed across 29 autosomal chromosomes and a heritability of 0.40

File S10: QMSim seed file for replicates 2-5 of the simulated data for a trait with 500 QTL distributed across 29 autosomal chromosomes and a heritability of 0.40

File S11: QMSim seed file for all 5 replicates of the simulated data for a trait with 2,000 QTL distributed across 29 autosomal chromosomes and a heritability of 0.10

File S12: QMSim seed file for all 5 replicates of the simulated data for a trait with 2,000 QTL distributed across 29 autosomal chromosomes and a heritability of 0.40

Table S1: Summary statistics of allelic substitution effects and genetic variance contribution for selected QTL within different QTL groups (heritability=0.1)

Table S2: Mean and standard deviation of F_{ST} scores for SNP windows surrounding QTL categorized by genetic variance contribution across different simulation scenarios (heritability= 0.10)
