# Supplementary material for: FST-Based Marker Prioritization Within Quantitative Trait Loci Regions and Its Impact on Genomic Selection Accuracy: Insights from a Simulation Study with High-Density Marker Panels for Bovines
Source: Genes (Basel). 2025 May 10;16(5):563. doi: 10.3390/genes16050563 (PMC12111557; doi:10.3390/genes16050563)
Supplement: Supplementary file 1 [file genes-16-00563-s001.zip › Table_S1.pdf]

QTL group[1]	# Simulated QTL	# Selected QTL	Allele substitution		Variance explained (%)	
			Mean	SD	Mean	SD
**Top 5%**	500	25	0.0535	0.009	1.101	0.362
	2000	100	0.0265	0.0036	0.297	0.096
**Q25_Q75**	500	250	0.0157	0.0051	0.100	0.064
	2000	1000	0.0078	0.0027	0.024	0.016
**Botton 5%**	500	25	0.0010	0.0004	0.0004	0.00025
	2000	100	0.0004	0.0002	0.00006	0.00005

[1] **Top 5%**= the 5% QTL with the largest effect; **Q25_Q75**= all QTL with effect between the 25 and 75% quantiles of the effect distribution; and **Bottom 5%**= the 5% QTL with the smallest effects.
