# Supplementary material for: FST-Based Marker Prioritization Within Quantitative Trait Loci Regions and Its Impact on Genomic Selection Accuracy: Insights from a Simulation Study with High-Density Marker Panels for Bovines
Source: Genes (Basel). 2025 May 10;16(5):563. doi: 10.3390/genes16050563 (PMC12111557; doi:10.3390/genes16050563)
Supplement: Supplementary file 1 [file genes-16-00563-s001.zip › Table_S2.pdf]

Scenarios[1]	Large QTL 95% quantile[2]		Medium QTL 25-75% quantiles		Small QTL 5% quantile	
	**Mean**	**SD**	**Mean**	**SD**	**Mean**	**SD**
**W1Q1**	0.00067	0.00047	0.00035	0.00030	0.00031	0.00024
**W2Q1**	0.00060	0.00054	0.00034	0.00037	0.00030	0.00025
**W3Q1**	0.00053	0.00056	0.00033	0.00036	0.00032	0.00032
**W4Q1**	0.00046	0.00054	0.00034	0.00040	0.00031	0.00034
**W1Q2**	0.00038	0.00027	0.00035	0.00029	0.00033	0.00028
**W2Q2**	0.00037	0.00032	0.00035	0.00034	0.00032	0.00032
**W3Q2**	0.00037	0.00038	0.00035	0.00038	0.00032	0.00036
**W4Q2**	0.00036	0.00042	0.00034	0.00041	0.00033	0.00039

[1] **Window size**: **W1**=50 SNPs, **W2**=100 SNPs, **W3**=200 SNPs, and **W4**=400 SNPs; total number of QTL: **Q1**=500 QTL, and **Q2**=2,000 QTL; [2] QTL effects were categorized based on their contribution to the genetic variance
